# Customized exogenous ferredoxin functions as an efficient electron carrier

**DOI:** 10.1186/s40643-021-00464-5

**Published:** 2021-11-03

**Authors:** Zhan Song, Cancan Wei, Chao Li, Xin Gao, Shuhong Mao, Fuping Lu, Hui-Min Qin

**Affiliations:** 1 Key Laboratory of Industrial Fermentation Microbiology of the Ministry of Education, Tianjin University of Science and Technology, Tianjin, 300457 People’s Republic of China; 2 College of Biotechnology, Tianjin University of Science and Technology, Tianjin, 300457 People’s Republic of China; 3 National Engineering Laboratory for Industrial Enzymes, Tianjin University of Science and Technology, Tianjin, 300457 People’s Republic of China

**Keywords:** Ferredoxin, Electron transfer, Electron bifurcation, [2Fe–2S] clusters

## Abstract

Ferredoxin (Fdx) is regarded as the main electron carrier in biological electron transfer and acts as an electron donor in metabolic pathways of many organisms. Here, we screened a self-sufficient P450-derived reductase PRF with promising production yield of 9OHAD (9α-hydroxy4-androstene-3,17-dione) from AD, and further proved the importance of [2Fe–2S] clusters of ferredoxin-oxidoreductase in transferring electrons in steroidal conversion. The results of truncated Fdx domain in all oxidoreductases and mutagenesis data elucidated the indispensable role of [2Fe–2S] clusters in the electron transfer process. By adding the independent plant-type Fdx to the reaction system, the AD (4-androstene-3,17-dione) conversion rate have been significantly improved. A novel efficient electron transfer pathway of PRF + Fdx + KshA (KshA, Rieske-type oxygenase of 3-ketosteroid-9-hydroxylase) in the reaction system rather than KshAB complex system was proposed based on analysis of protein–protein interactions and redox potential measurement. Adding free Fdx created a new conduit for electrons to travel from reductase to oxygenase. This electron transfer pathway provides new insight for the development of efficient exogenous Fdx as an electron carrier.

**Graphical Abstract:**

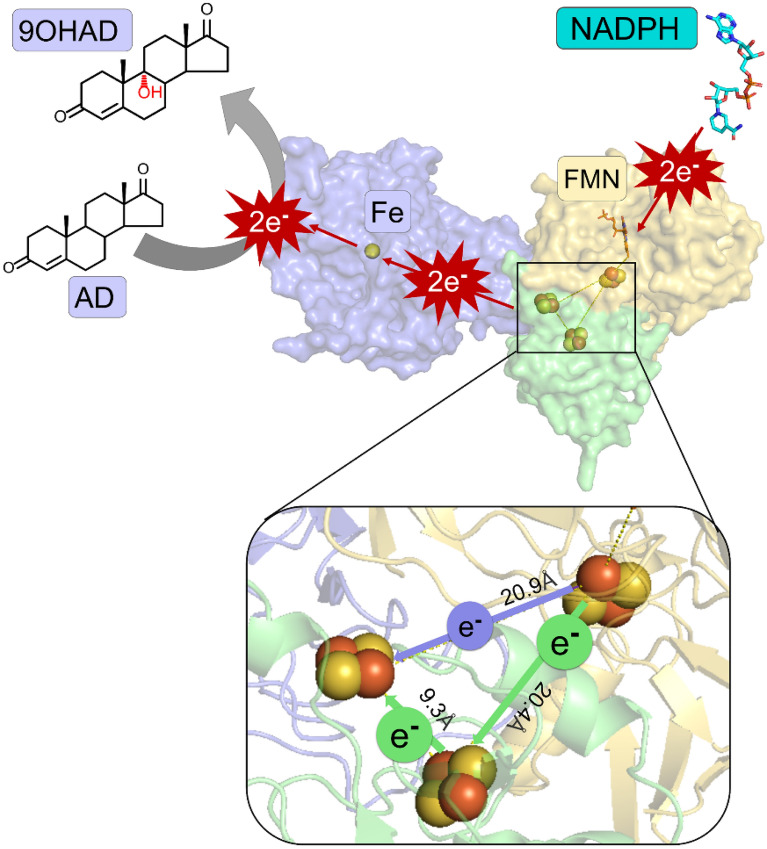

**Supplementary Information:**

The online version contains supplementary material available at 10.1186/s40643-021-00464-5.

## Introduction

Ferredoxin (Fdx) was firstly discovered and isolated from *Clostridium pasteurianum* (a non-photosynthetic anaerobic bacteria) by Mortenson et al. in 1962 (Mortenson et al. 1962). It is a small metalloprotein with [2Fe–2S] clusters as a redox center (Arnon 1988), they play a key role as electron carriers in various metabolic processes (Huang et al. 2016). In non-photosynthetic organisms, electron transfer is mediated by NAD(P)H (Arnon 1965; Goss et al. 2014; Martı´nez-Espinosa et al. 2003). The Fe–S cluster in Fdx physiologically transfers only one electron from NAD(P)H, and the reduced Fdx can drive the reaction more strongly because its redox potential is lower than that of NAD(P)H (Kameya et al. 2020). Fdx has been classified as [2Fe–2S], [4Fe–4S], [3Fe–4S] and [7Fe–8S] (Aono et al. 1992; Imai et al. 2001; Lovgreen et al. 2011; Ricagno et al. 2007), in which [2Fe–2S] clusters are divided into two types: plant-type and Rieske-type. In plant-type Fdxs, the [2Fe–2S] clusters are organized as [2Fe–2S] tetrahedrons, containing two inorganic Fe atoms and sulfurs from four conserved cysteine residues. In Rieske-type Fdxs, they are composed of inorganic iron atoms, two cysteine and histidine residues. Importantly, plant-type Fdx plays a central role in the electron transfer between the photosynthesis chain and various metabolic pathways (Böhme 1977; Kimata-Ariga et al. 2006; Lee et al. 2011).

Physiologically, except for the self-sufficient P450s with a fused Fdx or reductase domain, multiple Fdx/FNR is required for bacterial P450s in cells (Chen et al. 2020; Li et al. 2020). Previous researches indicated that Fdx and Fdx-NADP^+^-reductase (Fdx/FNR) could form complex during electron transfer process (Böhme 1977; Hurley et al. 1997; Kimata-Ariga et al. 2006; Lee et al. 2011; Quaranta et al. 2016). Fdx/FNR served as electron transfer intermediates in steroidal conversion catalyzed by P450s, so it is vital to find a suitable redox partner to carry out electron transfer with high efficiency (Kimata-Ariga et al. 2007; Quaranta et al. 2016; Ueno et al. 2013). Studies have established the principle of customizing non-physiological Fdx to support native-like P450 activity (Bell et al. 2012). The exogenous [2Fe–2S] domain was integrated into the N-terminus of KshB (Fdx reductase of 3-ketosteroid-9-hydroxylase) or TDO (toluene 2,3-dioxygenase reductase) was also reported for efficiently strengthening electron transfer by [2Fe–2S] cluster (Zhu et al. 2020). The electron transfer pathway from NADH to the terminal active-site mononuclear iron via FAD was proposed and the molecular mechanism of the redox-dependent interaction between NADH-dependent reductase and Rieske-type oxygenase was elucidated (Zhu et al. 2020). The structures of TDO including TDO-R (3EF6) and TDO-F (3DQY), and BphA4–BphA3 complex (FAD-NADH containing Fdx reductase BphA4 and Rieske-type [2Fe–2S] Fdx BphA3 in the electron transfer system of biphenyl dioxygenase BphA) were reported previously (Senda et al. 2007). The reductase BphA4 receives two electrons from NADH and the reduced BphA4–BphA3 complex then transfers an electron to the [2Fe–2S] clusters of two Fdx molecules through redox-dependent transient interactions. The reduced Fdx transfers the electron to the active center of the oxygenase to support the activation of dioxygen for biphenyl dihydroxylation. Self-sufficient P450 monooxygenases are highly active and convenient systems without partner reductase (Chen et al. 2020). The crystal structure of the self-sufficient P450 enzyme CYP116B46 has indicated that it contains the cofactor FMN to capture electrons from NADH and RP Fdx, which has most efficient electron transfer. The direction of electron transfer NADH → FMN → [2Fe–2S] → heme correlates with the domain arrangement, which undoubtedly provides a great help for the screening of redox partners (Zhang et al. 2020). Besides, based on the flavin-based electron bifurcation (FBEB) theory, the electron bifurcation phenomenon is formed by the oxidation of NADH in the oxidoreductase complex (Demmer et al. 2015; Duan et al. 2020; Lubner et al. 2017; Schut et al. 2019; Tokmina-Lukaszewska et al. 2018; Yuly et al. 2019). The FBEB bifurcation reaction forms a highly unstable flavonoid anion semiquinone (ASQ) to reduce Fdx (Kremp et al. 2020; Sucharitakul et al. 2020). The key step is to transfer electrons from a molecule with a medium reduction potential to one with lower potentials (thermodynamically unfavorable) by coupling the electrons with the transfer to the high potential acceptor (thermodynamically favorable) (Miller et al. 2019; Muller et al. 2018; Schut et al. 2019). Fdx is the electron carrier requiring for all reactions catalyzed by electron bifurcation proteins (Huang et al. 2016; Liang et al. 2019).

Hydroxylated steroids 9OHAD (9α-hydroxy-4-androstene-3,17-dione, an important precursor for the synthesis of glucocorticoid drugs) can be synthesized from AD (4-androstene-3,17-dione) by 3-ketosteroid 9α-hydroxylase (Ksh) (Fernandes et al. 2003; Zhu et al. 2020). Here, we screened ferredoxin reductases with plant-type and Rieske-type [2Fe–2S] clusters. A promising reductase of self-sufficient P450 CYP116B46 (V465-L779, named PRF, involving the flavin mononucleotide (FMN)-binding domain and [2Fe–2S] clusters) from *Tepidiphilus thermophiles* was expressed and purified*.* After elucidating the indispensable role of [2Fe–2S] clusters in the electron transfer process, we added the independent Fdxs to the reaction system to improve the conversion rate of AD to 9OHAD. A novel electron transfer pathway was proposed using exogenous Fdx as an efficient electron carrier based on analysis of protein–protein interactions and redox potential measurement. As for the mentioned ferredoxins, the structures of SyFdx (Ferraro et al. 2007), PrFdx (Nam et al. 2005), PsFdx1 (Senda et al. 2007) and TeFdx (Mutoh et al. 2015) and their functions as a redox partner have been reported. DmFdx1 and DmFdx2 transfer the electron to the active center of the oxygenase to support the activation of dioxygen for biphenyl dihydroxylation (Marelja et al. 2018; Palandri et al. 2015). However, their structures have not been reported.

## Materials and methods

### Plasmids construction, expression and purification of proteins

The coding sequences for the reductase domain and Fdx (V465-L779) of *Tepidiphilus thermophilus* CYP116B46 (PRF) (WP_055423153.1), *Thermosynechococcus elongatus*

BP-1 Fdx (TeFdx) (WP_011056851.1), *Drosophila melanogaster* Fdx1 (DmFdx1) and Fdx2 (DmFdx2) (BI626767.1 and BI168285.1), *Pseudomonas resinovorans* Fdx (PrFdx) (WP_011077882.1), *Pseudomonas sp.* Fdx (PsFdx1) (WP_015014864.1), *Sphingobium yanoikuyae* Fdx (SyFdx) (WP_025548169.1), were synthesized by GENEWZ (Suzhou, China) and cloned into the vector pET28a (+) between the *Nde*I and *Eco*RI restriction sites. KshA (Rieske-type oxygenase of 3-ketosteroid-9-hydroxylase) from *Rhodococcus erythropolis*, KshB (a Fdx reductase of 3-ketosteroid-9-hydroxylase) from *Mycobacterium tuberculosis* H37Rv, TDO_R (toluene 2,3-dioxygenase) from *Pseudomonas putida* are kept in the laboratory. The recombinant plasmid was introduced into *Escherichia coli* strains JM109 and BL21(DE3) were purchased from Novagen (Madison, WI, USA) as the host organism for gene cloning and protein expression, respectively. The strains, plasmids, and primers used in this study are listed in Additional file 1: Tables S1.

Bacteria were grown with shaking in lysogeny broth medium containing 40 μg/mL kanamycin at 37 °C. When the OD_600_ reached 0.6–0.8, 0.1 mM isopropyl-β-d-thiogalactopyranoside (IPTG) was added to induce the overexpression of proteins at 16 °C for 16–18 h. Then the bacteria were collected by centrifugation at 5000×*g* for 15 min at 4 °C, and resuspended in the lysis buffer (20 mM Tris–HCl, 40 mM imidazole, 0.5 M NaCl, 1 mM DTT, pH 7.4), lysozyme can be added to improve cell disruption. After ultrasonic disruption, the supernatant was collected by centrifugation at 40,000×*g* for 30 min at 4 °C and the His_6_-tagged proteins were trapped on Ni-NTA Superflow resin (Qiagen, Hilden, Germany). The proteins were eluted by elution buffer (20 mM Tris–HCl, 400 mM imidazole, 0.5 M NaCl, 1 mM DTT, pH 7.4), and dialyzed against 20 mM HEPES, pH 7.4 finally. The fractions were further purified using anion exchange chromatography on a Resource Q column (column volume: 6 mL, flow rate: 4 mL/min, GE Healthcare, Stockholm, Sweden).

### Activity assays

The NADH reduction activity was monitored by following UV absorption at 340 nm using a multimode plate reader (SpectraMax i3x, Molecular Devices, Silicon Valley, CA, USA). The specific operation method can refer to our existing research (Zhu et al. 2020). The Michaelis–Menten constants (*K*_m_), turnover number (*k*_cat_), catalytic efficiencies (*k*_cat_/*K*_m_) for each substrate were calculated by the Michaelis–Menten function in the GraphPad Prism (GraphPad Software, San Diego, CA, USA).

### Multienzyme cascade catalysis in vitro

The cell-free multi-enzyme catalysis was performed and mixing with 50 mM PBS (pH 7.4), 200 μM AD dissolved in 2% methanol, 300 μM NADH, and proteins (5 μM reductases, 5 μM Fdxs, and 2.5 μM KshA). The reaction of multi-enzyme cascade catalysis in vitro at room temperature, on a plate shaker at 500 rpm. The mixture was extracted with NaOH, dried by a nitrogen blower, and reconstituted with 100% methanol. The HPLC was used to determine the yield of 9OHAD (Zhu et al. 2020). The strains used are listed in Additional file 1: Tables S1, plasmids and primers used in this study are listed in Additional file 1: Tables S2.

### Site-directed mutagenesis

All variants described in this work were generated using the KOD-Plus Site-Directed Mutagenesis Kit (Toyobo, Japan) and the pET-28a (+) or pCold I vector harboring the wild-type sequence. The oligonucleotides designed to introduce the mutations are depicted in Additional file 1: Table S2. All mutations were confirmed by DNA sequencing. The genes of [2Fe–2S] clusters domain from KshB, TDO and PRF were truncated by PCR with the primers in Additional file 1: Table S2. The PCR product was digested with *Nde*I and *Eco*RI and reconstructed to generate plasmid △KshB, △TDO and △PRF. The sites of C314/C344 from KshB, H473/H493 from TDO, and C302/C332 from PRF were selected to construct mutants MKshB (C314H/C344H), MTDO(H473C/H493C) and MPRF(C302H/C332H) using the above Kit.

### Spectroscopic absorption

All UV–Vis spectroscopic absorption experiments were performed with Agilent Cary 2500 UV–Visible Spectrophotometer (Agilent Technologies, Beijing, China) at 25 ºC. The proteins were dialyzed against the same buffer (20 mM HEPES, pH 7.4). The spectral absorbance of 20 mM HEPES buffer was scanned in the range of 300–800 nm as a blank control, and then the UV–Vis spectral absorbances of the oxidized state proteins were measured. 100 mM sodium dithionite was added into the samples until there was no further decrease in absorbance in the 300–800 nm range and the spectrum of the reduced state proteins was measured.

### Homology modeling

A three-dimensional (3D) homology model were constructed using automodel command in Modeller 9.9.2, with the crystal structure of *Rhodococcus rhodochrous* 3-ketosteroid-9-alpha-hydroxylase 5 (PDB ID: 4QDC, 75.38% sequence identity with KshA), *Haloarcula marismortui* Fdx (PDB ID: 1DOI, 32.14% sequence identity with KshB-Fdx), human Fdx 2 (PDB ID: 2Y5C, 63.21% sequence identity with DmFdx1), and urate oxidase (PDB ID: 3PLM, 61.74% sequence identity with DmFdx2) as the template. The DOPE assessment scores were used to choose the best model. The model structures were visualized by PyMOL software (http://www.pymol.org).

### Protein–protein docking

All protein–protein docking used the website http://zdock.umassmed.edu/. The oxygenase–reductase complex was first obtained. The Fdx was then docked to the oxygenase–reductase complex, and obtained the best results by manually analysis the best top 10 results obtained on website http://zdock.umassmed.edu/. The subsequent processing and analysis are carried out using the PyMOL software (http://www.pymol.org).

### ITC measurement

The affinity of ferredoxin DmFdx2 with oxygenase KshA and reductase PRF was determined by Isothermal Titration Calorimetry 200 instrument (MicroCal, USA) at 25 °C. Each sample solution was dialyzed against the same buffer (20 mM HEPES, pH 7.4) and degassed under vacuum before calorimetry. The reaction cell and injection syringe were filled with 0.02 mM KshA/PRF and 0.2 mM DmFdx2, respectively. The reaction temperature, stirring speed, and reference power were set at 25 °C, 800 rpm, and 5.00 μcal/s, respectively. An aliquot comprising 2 μL of the DmFdx2 solution was added per injection, and each injection cycle lasted for 120 s. The interval between two injections was 60 s to allow for the signal to return to the baseline. The data were analyzed using Origin 7.0 software, and the data were fitted to the Wiseman equation using a one-site binding model. The stoichiometry (*n*), binding constant (*K*_*D*_), binding enthalpy (△*H*), and entropy change (△*S*) could be determined. The binding Gibbs energy change, △*G*, is calculated from the equation △*G* = − *RT* ln *K*_*D*_.

### Redox potential measurement

The electrochemical titration experiments were performed using Mettler T50 Automatic Potentiometric Titrator with an Ag/AgCl electrode at 25 °C. The 25 µM protein samples were dispersed in Tris–HCl buffer at pH 7.4. Sodium dithionite (100 mM) was used as the reducing agent. All solutions were degassed under vacuum with argon. The plug and play (PnP) burette with chip and the Ag/AgCl electrode were selected. Before the measurement, the burette was washed by pump ultrapure water under automatic cleaning mode program. In setting interface parameter, the titrant of sodium dithionite, the stirrer speed of 50%, and the sample volume of reducing agent were set to 10 mL. 10 mL protein sample was added to the titration cup. When the potential remained stable, sodium dithionite was titrated. The reduction potential and the volume of reducing agent were recorded when the potential was no longer changed.

### Construction of whole-cell catalysts system

Whole-cell catalysts [*E. coli* BL21 (DE3)] with different combinations of pET28a- and pETDuet-derived vectors bearing genes encoding KshA, PRF, and Fdx were used to convert AD to 9OHAD. To construct strain BLA-PD, the *PRF* gene was amplified, digested, and inserted into MCS1 site of pETDuet between *Eco*RI and *Hin*dIII sites, the gene encoding DmFdx2 was inserted into MCS2, between *Nde*I and *Xho*I, which created pETDuet-PRF-DmFdx2. pETDuet-PRF-DmFdx2 and pET28a harboring *KshA* were co-transformed into *E. coli* BL21(DE3) cells. The similar operation mode was used to construct strain BLA-PP, in which the *PRF* and *PsFdx1* genes were inserted into pETDuet and created pETDuet-PRF-PsFdx1. pETDuet-PRF-PsFdx1 and pET28a harboring *KshA* were co-transformed into *E. coli* BL21(DE3) cells. The control strain of BLA-P was constructed using pET28a-KshA and pCold I-PRF, and co-transformed into *E. coli* BL21(DE3) cells. The constructed strains are listed in Additional file 1: Table S1 and plasmids are listed in Additional file 1: Table S2.

### Bioconversion of AD to produce 9OHAD

The constructed recombinant strains are listed in Additional file 1: Table S1. The plasmid construction and protein expression operation are the same as described above. The cells were harvested by centrifugation at 5000×*g* for 10 min and resuspended in 30 mL 50 mM PBS (pH 7.4). The production of 9OHAD was conducted in reaction mixture containing 50 g/L wet cells, 5 g/L AD dissolved in 2% methanol and 22.8 g/L methyl-β-cyclodextrin, at 35 °C for 5 h (Zhu et al. 2020). The products were determined by HPLC.

## Results and discussion

### Comparison of reductases for hydroxylation reaction toward AD

**Fig. 1:**
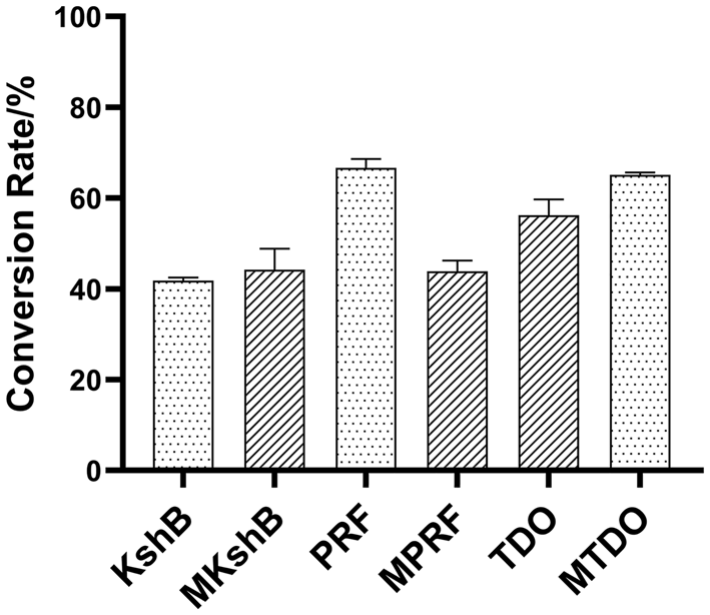
Conversion rate of AD to 9OHAD. AD conversion rate of various reductases of KshB, PRF, TDO or mutants MKshB, MPRF, MTDO in KshA + reductase catalytic reaction system

The reductases of KshB and TDO have been screened previously with AD as the substrate (Zhu et al. 2020). A novel reported reductase of the self-sufficient P450 enzyme CYP116B46 from *Tepidiphilus thermophilus* (named PRF), which contains plant-type [2Fe–2S] cluster, was first mined for bioconversion of AD. We further investigated the catalytic efficiency of PRF in both NAD(P)H reduction activity and 9OHAD reaction system based on research on KshB and TDO (Table 1). By comparing the reduction activity toward NADH, we found that PRF showed higher activity with *k*_cat_/*K*_m_ of 1.70 s^−1^ μM^−1^ than TDO (0.43 s^−1^ μM^−1^) and KshB (0.34 s^−1^ μM^−1^). Furthermore, PRF also showed the higher AD conversion rate of 68.4% than TDO (54.8%) and KshB (42.0%) (Fig. 1; Additional file 1: Table S3). Therefore, PRF showed the promising potential as reductase partner of KshA during hydroxylation of steroidal substrates than KshB or TDO.

**Table 1:** Kinetic parameters of reductases and ferredoxin toward NADH

Reductase	*K*_m_ (μM)	*k*_cat_ (s^−1^)	*k*_cat_/*K*_m_ (s^−1^ μM^−1^)
TDO^a^	65.37 ± 2.71	28.21 ± 0.91	0.43
△TDO	/	/	/
MTDO	63.53 ± 2.95	34.27 ± 0.69	0.54
KshB^a^	70.85 ± 4.08	23.88 ± 1.06	0.34
△KshB	/	/	/
MKshB	69.94 ± 2.12	24.13 ± 0.89	0.35
PRF	39.11 ± 1.49	66.43 ± 0.34	1.70
△PRF	/	/	/
MPRF	63.12 ± 2.37	31.27 ± 1.17	0.50

Data are shown as the mean ± SD from three independent experiments

^a^Data from Zhu et al.

### Activity analysis of reductase with two types of [2Fe–2S] clusters

**Fig. 2:**
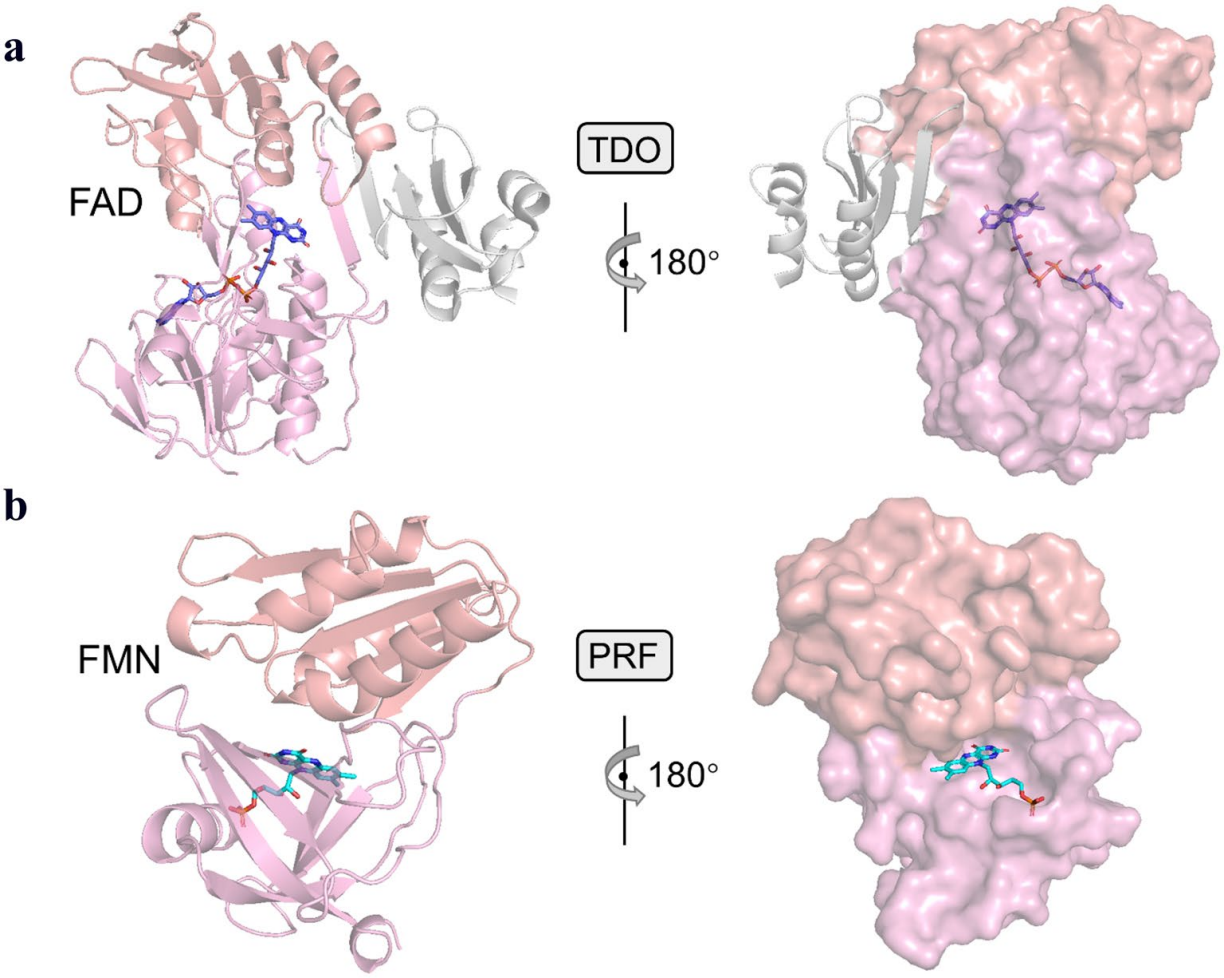
Structural comparison of reductase domain of TDO and PRF. The cartoon and surface representation of the reductase domain of TDO (**a**) and PRF (**b**). NADH-binding domain is shown in sallow and FAD/FMN-binding domain is shown in pink, FAD and FMN are represented by sticks

We compared the crystal structures of two reductases TDO-R (PDB ID: 3EF6) with PRF (PDB ID: 6LAA). Structural analysis revealed that both TDO and PRF contained NAD(P)H domain, and their flavin-binding motifs belonged to different types: FAD-binding and FMN-binding (Fig. 2). FAD captured electrons from NADH and transfers to [2Fe–2S] cluster Fe atom of TDO-Fdx (Friemann et al. 2009). The structural comparison showed that the TDO contained an extra domain (gray part). In previous research, a “swing domain model” of P450BM3 FMN-binding domain operated the back-and-forth motions, which was governed by a flexible hinge domain (Chen et al. 2020). That implied that TDO-Fdx might swing to a favorable position to carry out the effective electron transfer from FAD to [2Fe–2S] cluster (Fig. 2a). In PRF, FMN captured electrons from NADH and transferred the electrons from the methyl group of C8 to Fe atom of [2Fe–2S] cluster (Fig. 2b) (Zhang et al. 2020). The truncated [2Fe–2S] clusters of the oxidoreductases (△KshA, △KshB, △TDO, △PRF) had no longer exhibited catalytic activity toward AD. It indicated that [2Fe–2S] clusters played the irreplaceable role during the hydroxylation reaction of AD (Table 1). Importantly, the structures of [2Fe–2S] clusters in TDO, KshB and PRF are also different, which may result in completely different catalytic activities and conversion rate. The [2Fe–2S] cluster of TDO-F is the Rieske-type (Fe ligated by 2Cys and 2His), while PRF and KshB are the plant-type (Fe ligated by 4Cys).

To further investigate the importance of [2Fe–2S] clusters in reductases, we compared the catalytic activity of various reductases containing two different types of [2Fe–2S] clusters. Besides, we mutated the TDO to MTDO (plant-type) (Additional file 1: Table S1). Meanwhile, KshB and PRF were mutated to MKshB and MPRF (Rieske-type) (Additional file 1: Table S1), respectively. After mutation, the NADH reduction activity *k*_cat_/*K*_m_ of MTDO increased from 0.43 s^−1^ μM^−1^ to 0.54 s^−1^ μM^−1^. KshB showed the same activity (0.34 s^−1^ μM^−1^) with MKshB (0.35 s^−1^ μM^−1^). Surprisingly, MPRF showed decreased activity from 1.70 s^−1^ μM^−1^ to 0.50 s^−1^ μM^−1^ (Table 1). The data of AD conversion rate of three mutants also showed the same tendency with NADH reduction activity that mutated MPRF also decreased the AD conversion rate (Additional file 1: Table S3). These results implied that plant-type cluster seems to have higher electron transfer efficiency than Rieske-type cluster.

**Fig. 3:**
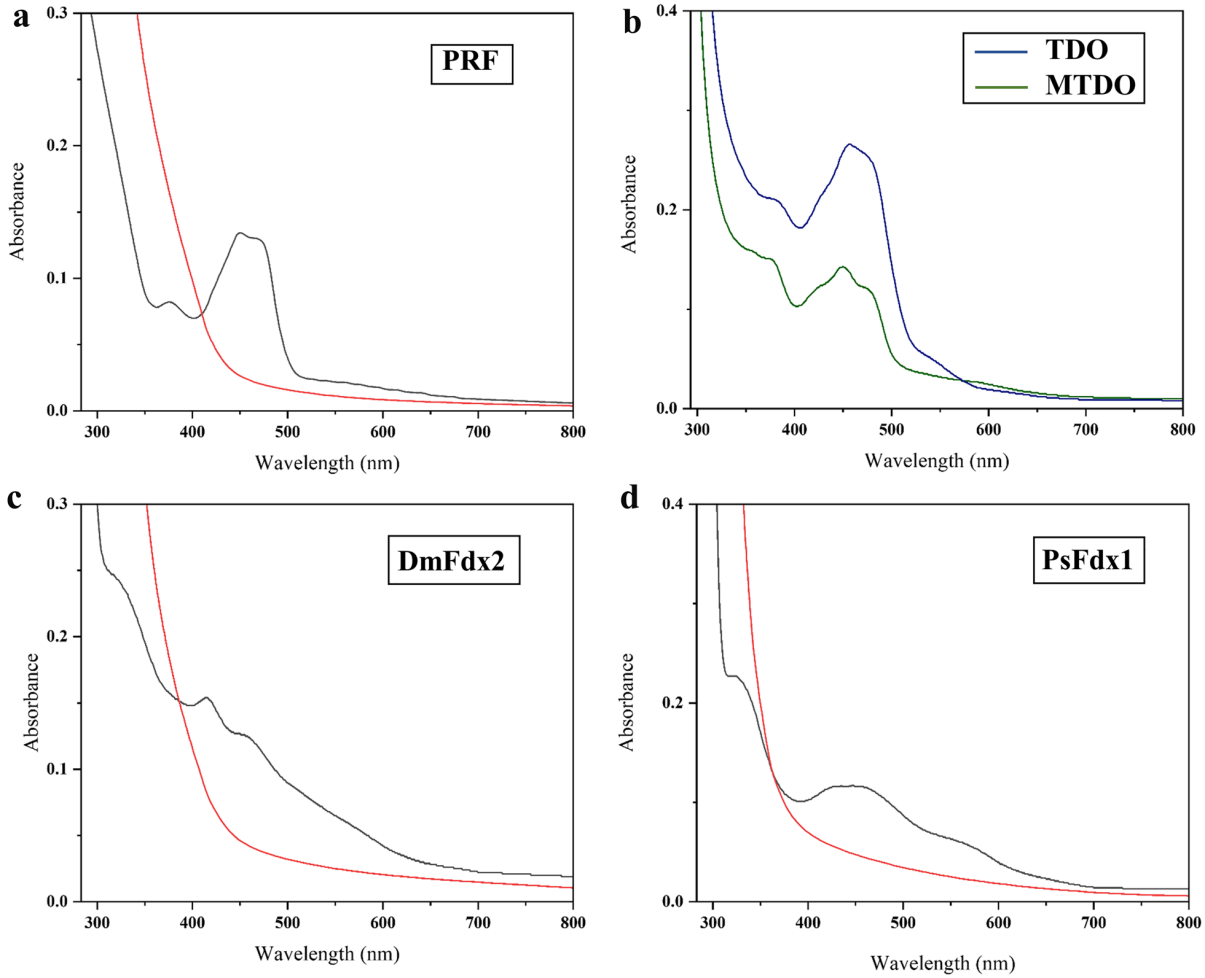
UV–Vis absorption spectrum of Fdxs and reductase. The UV–Vis absorption spectra of the oxidized state and sodium dithionite-reduced are black and red, respectively, and shown as **a** reductase PRF, **c** DmFdx2 (plant-type) and **d** PsFdx1 (Rieske-type). **b** Comparison of the UV–Vis absorption spectra of wild-type TDO (blue) and mutant MTDO (green)

More importantly, the UV–vis absorption spectra (Fig. 3a, b) were typical characteristics of flavoproteins. Plant-type PRF (Fig. 3a) and MTDO (Fig. 3b) had almost identical UV–Vis spectra, with three main absorption peaks at 378, 447 and 475 nm. In contrast, the wild-type TDO (Rieske-type) also showed the same main absorption peaks at 378 and 447 nm, but a slight bathochromic shift of the band at 475 nm and a relative amplitude of the 530–550 nm shoulder (Fig. 3b). This indicated that the modification of TDO (Rieske-type) to MTDO (plant-type) resulted in the slight changes in the prosthetic group environment. These small changes implied that the electronic structure of Rieske-type and plant-type [2Fe–2S] cluster was different and hence of different interactions between the two types of [2Fe–2S] clusters and the protein.

### Screening of free Fdx proteins for enhancement of steroidal conversion

The [2Fe–2S] clusters have been testified to be important for catalytic activities. To verify whether free Fdx functions as electron transfer intermediate, we investigated the effects of two types of free Fdxs (Rieske-type and plant-type) according to the conversion of AD to 9OHAD using multi-enzyme catalytic system in vitro (KshA + PRF) (Additional file 1: Fig. S1, Table S4). Six Fdxs genes were screened as candidates namely PsFdx1, SyFdx, PrFdx (Rieske-type Fdxs) and TeFdx, DmFdx1, DmFdx2 (plant-type Fdxs).

After adding various free Fdxs in the 9OHAD catalytic reaction system containing reductase PRF and oxygenase KshA, the AD conversion rate were determined and the Fdxs candidate proteins were evaluated. The most outstanding in six Fdxs are Rieske-type PsFdx1 and plant-type DmFdx2 (Additional file 1: Fig. S1). Therefore, we selected these two Fdxs for further bioconversion of AD to 9OHAD in oxidoreductase system using reductases KshB, TDO and PRF. In particular, when adding Rieske-type Fdx PsFdx1 into the catalytic reaction system, the AD conversion rates of KshA + PRF + PsFdx1 and KshA + PRF + DmFdx2 were 80.6% and 85.2% (Additional file 1: Fig. S2a), which were 1.18- and 1.25-fold higher than only using KshA + PRF (68.4%), respectively. The reaction systems of TDO and KshB also showed the similar increase of AD conversion rates when adding two types of Fdx (Additional file 1: Fig. S2b, c). When free Fdx was added to the catalytic reaction system, the electron transfer was effectively promoted due to the mediation of free Fdx among reductase and oxygenase. Furthermore, the plant-type DmFdx2 was more efficient than Rieske-type PsFdx1, when adding these two different kinds of Fdxs to catalytic reaction system.

### Structural analysis of two types of Fdxs

All the six free Fdxs are [2Fe–2S] clusters, which can be divided into plant-type and Rieske-type according to the difference of coordinating amino acids in the [2Fe–2S] cluster (Additional file 1: Fig. S3). Sequence alignment showed that all the Rieske-type Fdxs have conserved Cys-X1-His-segment-Cys-X2-His motif (Additional file 1: Fig. S4a). N-terminal portion of the Rieske-type Fdx consists of a Rieske [2Fe–2S] cluster domain, which consists of β strands and loops (Ferraro et al. 2007; Murzin et al. 1995). Four residues in the α subunit, 2His and 2Cys, coordinate the Rieske non-heme iron in [2Fe–2S] cluster. 2His coordinate one iron, while 2Cys coordinate the other (Additional file 1: Fig. S3a, b, c). At the same time, all the plant-type Fdxs have conservative Cys-X4/X5-Cys-X2-Cys-segment-Cys motif (Additional file 1: Fig. S4b). The C-terminal portion consists of a plant-type [2Fe–2S] cluster domain, which consists of loops, β strands and α spiral. Four residues of Cys in the α subunit coordinate two irons of non-heme iron cluster [2Fe–2S] (Additional file 1: Fig. S3d, e, f).

The UV–Vis spectroscopic absorption of all oxidized Fdxs showed characteristic absorption spectra in range of 300–800 nm. DmFdx2 (Fig. 3c) and PsFdx1 (Fig. 3d) showed distinguished different UV–Vis spectra. DmFdx2 showed typical spectrum of plant-type Fdxs with three main absorptions at 330, 415 and 465 nm. After reduction with sodium dithionite, the absorption decreased by 52% at 420 nm and 49% at 465 nm. The UV–vis absorption spectrum of Rieske-type PsFdx1 (Fig. 3b) showed bands at 330 and 450 nm, but a relative amplitude of the 550–580 nm shoulder. When reduced with sodium dithionite, the absorbance of PsFdx1 decreased in the entire UV–visible range. These typical features of two types [2Fe–2S] of Fdxs indicated that the electronic structure of Rieske-type and plant-type [2Fe–2S] cluster was different.

### Structural analysis of proposed electron transfer pathway

To analyze the electron transfer pathway between free Fdx, reductase and oxygenase, we targeted the reaction system of the KshA (oxygenase), PRF (reductase) and DmFdx2 (Fdx) to investigate the electron transfer pathway using protein–protein docking (Additional file 1: Fig. S5). Interestingly, the docked structure complex revealed that there were two potential electron transfer pathways existing in the reaction system. Adding free Fdx creates a new conduit for electrons to travel from reductase to oxygenase. In the KshA + PRF catalytic reaction system, the electron transfer pathway is PRF (NADH → FMN → [2Fe–2S]) → KshA ([2Fe–2S] → Fe). After free Fdx was added to the catalytic reaction system, an auxiliary electron transfer pathway of PRF(NADH → FMN → [2Fe–2S]) → DmFdx2 ([2Fe–2S]) → KshA ([2Fe–2S] → Fe) might be formed between PRF and DmFdx2, in which the electrons could also be transferred to the [2Fe–2S] of free Fdx, and then were captured by the [2Fe–2S] of the PRF. The physical distance between two [2Fe–2S] clusters of PRF and KshA (20.9 Å) was almost the same with the distance between [2Fe–2S] clusters of PRF and DmFdx2 (20.4 Å). It seems that electrons have the same probability to be transferred by both pathways (Additional file 1: Fig. S5). Furthermore, the distance between [2Fe–2S] clusters of DmFdx2 and KshA was only 9.3 Å, implied that electrons could be transferred more effectively to KshA. The structural information implied that two electron transfer pathways might be more efficient for improving the AD conversion rate.

### Analysis of protein–protein interactions identified by ITC

**Fig. 4:**
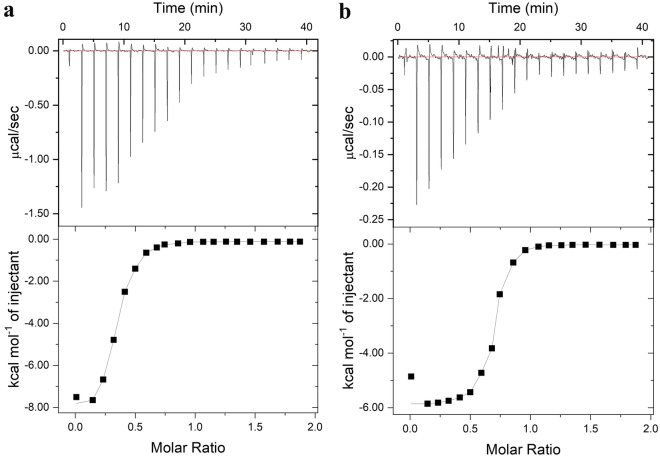
The affinity measurements between DmFdx2 and PRF/KshA by ITC. The affinity of reductase PRF (**a**) and oxygenase KshA (**b**) (0.2 mM DmFdx2 vs 0.02 mM PRF/KshA) to DmFdx2 was determined by ITC 200 in 20 mM HEPES buffer (pH 7.4) at 25 °C. The raw data of the calorimetric titration and the curve fit of the observed heat are displayed in the upper and lower panels, respectively

To further identify the proposed electron transfer pathway of free Fdx as an electron carrier, the interactions of DmFdx2 with KshA and PRF were measured via thermodynamics ITC experiments. The results of the calorimetric titrations of DmFdx2 to PRF (Fig. 4a) and DmFdx2 to KshA (Fig. 4b) are shown in Fig. 4. The thermodynamic parameters are collected in Table 2. In both cases, an exothermic heat pulse (Δ*H* < 0) was observed after each injection of DmFdx2 into the aptamer solution KshA and PRF. This indicated that the binding process was an exothermic reaction. However, the interaction of PRF with DmFdx2 was associated with a larger favorable enthalpy (△*H* = − 8 kJ mol^−1^) compared with KshA (△*H* = − 6 kJ mol^−1^). The values of the binding constants and the Gibbs energy changes indicated that the associations were strongly favored, at 25 °C, from a thermodynamic point of view. The binding constant for the interaction of KshA with DmFdx2 (*K*_D_ = 2.10 × 10^7^ M^−1^) is about 1.5-fold greater than that for the PRF–DmFdx2 interaction (*K*_D_ = 1.50 × 10^7^ M^−1^). The values of entropy change (△*S* < 0) showed that, in both cases, the binding processes were enthalpy driven. The conformational entropy changes are large favorable contributor to complexation. The increase in entropy helps stabilize the complex to a large extent (Lee et al. 2011). This indicated that after the initial attraction, the formation of the catalytically active complex was driven by the favorable entropy changes. All the thermodynamic data of ITC above indicated the rationality and efficiency of the proposed electron transfer pathway using exogenous Fdx as an effective electron carrier.

**Table 2:** Thermodynamic parameters for the interaction with DmFdx2

	*n*	*K*_*D*_ (M^−1^)	△*H *(KJ mol^−1^)	△*S *(KJ mol^−1^ K^−1^)	△*G*_298k_ (KJ mol^−1^)
PRF	0.75 ± 0.01	(1.50 ± 0.10) × 10^7^	− 8 ± 0.82	0.11 ± 0.02	− 40.94 ± 1
KshA	0.40 ± 0.02	(2.10 ± 0.10) × 10^7^	− 6 ± 0.13	0.12 ± 0.03	− 41.81 ± 1

### Analysis of redox potential and electron bifurcation

**Fig. 5:**
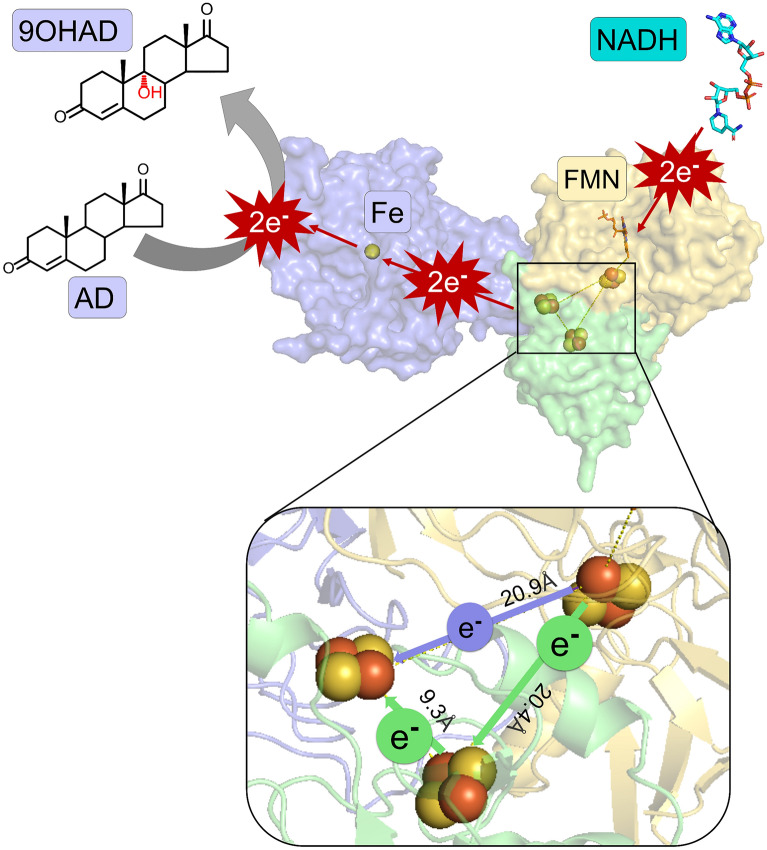
Analysis of the proposed electron transfer pathway between oxidoreductase and free Fdx. The overall structures of reductase PRF, free ferredoxin DmFdx2 and oxygenase KshA are shown as surface representation. NADH and FMN are shown as sticks, iron atoms and [2Fe–2S] cluster groups are shown as spheres

To clarify the auxiliary electron transfer pathway between two [2Fe–2S] clusters of reductase PRF and ferredoxin DmFdx2, we performed the measurement of the redox potential (Table 3). The redox potential measurement indicated that the single-pathway potential and the dual-pathway potential are different. Particularly, single-pathway showed the redox potential of PRF (− 365 mV) and KshA (− 346 mV). However, DmFdx2 had lower reduction potential (− 390 mV) and higher reduction ability in dual-pathway. It might be reasonable that the lower the free energy of the final state, the higher would be the electron transfer efficiency. After the PRF captures the two electrons from the FMN, one electron with a lower energy level is directly transferred to the oxygenase KshA, and the other electron with a higher energy level is transferred to reduce DmFdx2 (Fig. 5). Compared with the potential difference of PRF (− 365 mV) and KshA (− 346 mV), the larger potential difference between PRF (− 365 mV) and DmFdx2 (− 390 mV) has more energy and tendency to transfer electrons. Furthermore, the distance between the two [2Fe–2S] clusters of DmFdx2 and oxygenase KshA was only 9.3 Å, which seems to transfer electrons more effectively (Additional file 1: Fig. S5). The analysis of redox potential and electron bifurcation was consistent with the bioconversion of AD that adding Fdx into the catalytic system might improve the conversion rate (Table 3).

**Table 3:** Redox potential of reductase and ferredoxin

Proteins (V1)	Titrant (V2)	Volume ratio (V1:V2)	Electric potential (mV)
KshA	Na_2_S_2_O_4_	2:1	− 346 ± 1
KshB	Na_2_S_2_O_4_	2:1	− 352 ± 1
PRF	Na_2_S_2_O_4_	2:1	− 365 ± 2
DmFdx2	Na_2_S_2_O_4_	2:1	− 390 ± 1
KshB + KshA	DmFdx2	1:3	− 80 ± 3
PRF + KshA	DmFdx2	1:3	− 94 ± 1

The redox potentials also indicated that PRF (− 365 mV) has lower redox potential than KshB (− 352 mV). It implied that reductase PRF had stronger redox capacity and higher biotransformation capacity than KshB. In addition, by using free Fdx to titrate the mixed system of reductase and oxygenase, we found that the final titration states showed lower redox potential of PRF + KshA (− 94 mV) than KshB + KshA (− 80 mV). All these redox potential data explain excellent ability in steroid transformation of PRF + Fdx + KshA reaction system.

### Bioconversion of AD to produce 9OHAD

Whole-cell catalysis systems were constructed in the *E. coli* BL21(DE3) for stable and sustainable production of 9OHAD. We constructed three industrial strains of BLA-PD, BLA-PP and BLA-P. Whole-cell catalytic system without free Fdxs of BLA-P (KshA + PRF-BL21) only showed the yield of 9OHAD with 76.7% in 5 h. However, the whole-cell catalytic system with free Fdxs of BLA-PD (KshA + PRF + DmFdx2-BL21) showed the highest yield of 9OHAD with (95.4%) in 5 h, followed by BLA-PP (KshA + PRF + PsFdx1-BL21) (90.2%). This implied that add free Fdxs, especially plant-type DmFdx2, attributed to increase the electron transfer efficiency in the catalytic system of KshA + PRF. It also implied the importance of the predominant second electron transfer pathway mentioned above.

In comparison, the engineered *Mycobacterium neoaurum* ATCC 25795 reached a 54.5% yield of 9OHAD from 20 g/L phytosterol in 72 h as substrate by modifying key genes related to metabolism to increase cell permeability (Xiong et al. 2020). The *Rhodococcus erythropolis* RG1-UV29 strain reached a 93% yield of 9OHAD from 20 g/L AD in 24 h (Geize et al. 2001). However, the recombinant *E. coli* only reached a 63% yield of 9OHAD from 1 g/L AD in 48 h (Petrusma et al. 2009). Therefore, the constructed whole-cell catalytic system of BLA-PD with free Fdxs exhibited an excellent catalytic ability, and has the potential to be used industrially for efficient 9OHAD production.

In our previous research, Zhu et al. constructed *E. coli* BL21(DE3) strain of BLKA-RTM-F, which contained the NADH recycling system and a modified TDO mutant with two Rieske-type [2Fe–2S] clusters at the N-/C-terminus. It exhibited excellent 9OHAD productivity with 5.24 g/L from 5 g/L AD and a yield of 99.3% without by-products (Zhu et al. 2020). However, BLA-PD (KshA + PRF + DmFdx2-BL21) constructed in the present work showed the highest AD conversion rate of 95.4%, slightly lower than BLKA-RTM-F. It may be due to the construction of NADH recycling system in BLKA-RTM-F, because NADH could be completely consumed in about 16 min. It was thought to be a limiting factor. The future work would focus on the NADH recycling system combined with the current whole-cell catalysts to improve the AD conversion rate and sustainability.

## Conclusions

We screened the reductase PRF with high NADH reduction activity and AD conversion rate. The screened plant-type DmFdx2 could strengthen the biotransformation of AD due to the high electron transfer efficiency. Based on the electron bifurcation theory, we inferred that free Fdx created a new conduit for electrons to travel from reductase to oxygenase. The protein–protein structure docking and redox potential measurement had proven this speculation. The thermodynamic data of ITC also indicated the rationality of the new and efficient electron transfer method using exogenous Fdx as an effective electron carrier. Whole-cell catalytic system with free Fdxs of BLA-PD (KshA + PRF + DmFdx2-BL21) showed a 95.4% yield of 9OHAD indicating the importance of effective electron transfer carrier and a predominant reductase with high activity.

### Supplementary Information

Additional file 1: **Tables S1 and S2**: bacterial strains, primers, and plasmids used; **Tables S3**: AD conversion rate of reductase and ferredoxin; **Tables S4 and Figure S1**: AD conversion rate of six Fdxs in KshA + PRF + Fdx reaction system; **Figure S2**: conversion rate of AD in multi-enzyme cascade catalysis adding free Fdxs; **Figure S3**: structural comparison of plant-type and Rieske-type Fdxs; **Figure S4**: sequence alignment and phylogenetic tree analysis of [2Fe–2S] cluster domain of Fdxs and oxidoreductases; **Figure S5**: results of protein–protein docking.

## Data Availability

All the needed data are provided in the manuscript.

## Abbreviations

9OHAD: 9α-Hydroxy4-androstene-3,17-dione
AD: 4-Androstene-3,17-dione
KshA: Rieske-type oxygenase of 3-ketosteroid-9-hydroxylase
KshB: Plant-type reductase of 3-ketosteroid-9-hydroxylase
TDO: Toluene 2,3-dioxygenase reductase
PRF: Self-sufficient P450 CYP116B46 (V465-L779) from *Tepidiphilus thermophiles*
Fdx: Ferredoxin
TeFdx: *Thermosynechococcus elongatus* BP-1 Fdx
DmFdx1: *Drosophila melanogaster* Fdx1
DmFdx2: *Drosophila melanogaster* Fdx2
PrFdx: *Pseudomonas resinovorans* Fdx
PsFdx1: *Pseudomonas sp.* Fdx
SyFdx: *Sphingobium yanoikuyae* Fdx
