# Supplementary material for: Customized exogenous ferredoxin functions as an efficient electron carrier
Source: Bioresour Bioprocess. 2021 Nov 3;8(1):109. doi: 10.1186/s40643-021-00464-5 (PMC10992505; doi:10.1186/s40643-021-00464-5)
Supplement: Supplementary file 1 — Additional file 1: Tables S1 and S2: bacterial strains, primers, and plasmids used; Tables S3: AD conversion rate of reductase and ferredoxin; Tables S4 and Figure S1: AD conversion rate of six Fdxs in KshA + PRF + Fdx reaction system; Figure S2: conversion rate of AD in multi-enzyme cascade catalysis adding free Fdxs; Figure S3: structural comparison of plant-type and Rieske-type Fdxs; Figure S4: sequence alignment and phylogenetic tree analysis of [2Fe–2S] cluster domain of Fdxs and oxidoreductases; Figure S5: results of protein–protein docking. [file 40643_2021_464_MOESM1_ESM.docx]

**Supplementary Information**

**Customized exogenous ferredoxin functions as an efficient electron carrier**

Zhan Song^1^, Cancan Wei^1^, Chao Li^1^, Xin Gao^1^, Shuhong Mao^1,^*, Fuping Lu^1,^*, and Hui-Min Qin^1,^*

^1^ *Key Laboratory of Industrial Fermentation Microbiology of the Ministry of Education; Tianjin Key Laboratory of Industrial Microbiology; College of Biotechnology, Tianjin University of Science and Technology; National Engineering Laboratory for Industrial Enzymes; Tianjin 300457, P. R. China*

*Corresponding authors: College of Biotechnology, Tianjin University of Science and Technology

S. Mao: shuhongmao@tust.edu.cn;

F. Lu: lfp@tust.edu.cn;

H.-M. Qin: huiminqin@tust.edu.cn.

Tel: +86-22-60602949. Fax: +86-22-60602298

**Table S1.** Bacterial strains used in this study

| Strains | Description | Source |
| --- | --- | --- |
| *E. coli* JM109 | cloning | Lab stock |
| *E. coli* BL21 (DE3) | expression | Lab stock |
| KshA-BL21 | pET28a carries *KshA*; BL21 (DE3) | Lab stock |
| KshB-BL21 | pCold I carries *KshB*; BL21 (DE3) | Lab stock |
| TDO-BL21 | pET28a carries *TDO*; BL21 (DE3) | Lab stock |
| PRF-BL21 | pET28a carries reductase domain and ferredoxin of CYP116B46; BL21 (DE3) | This study |
| △KshA-BL21 | pET28a carries *KshA* deletion ferredoxin domain (M1-H143); BL21 (DE3) | This study |
| △KshB-BL21 | pCold I carries *KshB* deletion ferredoxin domain (P303-E359); BL21 (DE3) | This study |
| △TDO-BL21 | pET28a carries *TDO-R* gene; BL21 (DE3) | This study |
| △PRF-BL21 | pET28a carries *PRF* deletion ferredoxin domain; BL21 (DE3) | This study |
| MKshB-BL21 | pCold I carries KshB-C314/344H mutant | This study |
| MTDO-BL21 | pET28a carries TDO-H473/493C mutant | This study |
| MPRF-BL21 | pET28a carries PRF-C302/332H mutant | This study |
| TeFdx-BL21 | pET28a carries *TeFdx* gene; BL21 (DE3) | This study |
| DmFdx1-BL21 | pET28a carries *DmFdx1* gene; BL21 (DE3) | This study |
| DmFdx2-BL21 | pET28a carries *DmFdx2* gene; BL21 (DE3) | This study |
| PrFdx-BL21 | pET28a carries *PrFdx* gene; BL21 (DE3) | This study |
| PsFdx1-BL21 | pET28a carries *PsFdx1* gene; BL21 (DE3) | This study |
| SyFdx-BL21 | pET28a carries *SyFdx* gene; BL21 (DE3) | This study |
| GST-DmFdx2-BL21 | pGEX-6p-3 carries *DmFdx2* gene; BL21 (DE3) | This study |
| BLA-PD | pET28a carries *KshA* gene, pETDuet carries *PRF* and *DmFdx2*; BL21 (DE3) | This study |
| BLA-PP | pET28a carries *KshA* gene, pETDuet carries *PRF* and *PsFdx1*; BL21 (DE3) | This study |
| BLA-P | pET28a carries *KshA* gene; pET28a carries *PRF* gene; BL21 (DE3) | This study |

**Table S2.** Primers and plasmids used in this study.

| Primers and plasmids | Sequence of primer |
| --- | --- |
| primers |  |
| △KshA_F | GGAATTC**CATATG**GACCCGCAAGGCAATC |
| △KshA_R | **GAATTC**CGGTTAGCTCGTCGTTTCCAG |
| △KshB_F | GGAATTC**CATATG**ATGTTGACCGAGGCAATTGG |
| △KshB_R | **GAATTC**CGGTTACGCGTCC AGGCCC |
| △TDO_F | GGAATTC**CATATG**ATGGCCACCCATGTTGC |
| △TDO_R | **GAATTC**CGGTTACGGGCCC GGATTTTC |
| △PRF_F | GGAATTC**CATATG**GTGAGAACCATGGAAGTGG |
| △PRF_R | **GAATTC**CGGTTAACCAAAA AAATGTTCCA CTCTCAG |
| MKshB-C314H_F | GCGCATGCGTGCACC |
| MKshB-C314H_R | ACCGCAGTGGCCTTCC |
| MKshB-C344H_F | GCCCATCAATCTCGC |
| MKshB-C344H_R | CAAAATCAGT CCCTCATC |
| MTDO-H473C_F | ACCTGCGGCGACTG |
| MTDO-H473C_R | ACAAGTATCTTGCACTGCAA AG |
| MTDO-H493C_F | TTATGCTTTGGCAAATTCTG |
| MTDO-H493C_R | AGTGCATTCCACAATGTCG |
| MPRF-C302H_F | ACCCATGAAGTGAGTGTG |
| MPRF-C302H_R | GCCACACAGG CCTTC |
| MPRF-C332H_F | TGCCACTGCAGCAGAGC |
| MPRF-C332H_R | CATCATTCTTCTATTTTCTCTTCTTTCTG |
| TDO-Fdx_F | GGAATTC**CATATG**ATGACTTGGACCTATATTTTACG |
| TDO-Fdx_R | **GAATTC**CGGTTATTTCAGT TCACCGTTATC |
| PRF-Fdx_F | GGAATTC**CATATG**GAACCAAGCCATCTGGATC |
| PRF-Fdx_R | **GAATTC**CGGTTACAGATCC AGCACCAGTC |
| KshA-Fdx F | GGAATTC**CATATG**CATATGGCCCTC |
| KshA-Fdx_R | **GAATTC**CGGTTAATGCCAC ACG |
| KshB-Fdx_F | GGAATTC**CATATG**CCGTTCTCCTGC |
| KshB-Fdx_R | **GAATTC**CGGTTACTCGTCG TAGGTC |
| GST-DmFdx2_F | **GGATCC**CTGGTGATTAATAGTTGCCGTGC |
| GST-DmFdx2_R | **CTCGAG**TGCTGCGCGGGCATC |
| Plasmids |  |
| pET28a-KshA | pET28a, contain *KshA* gene (codon-optimized), Kan^R^ |

| pET28a-PRF | pET28a, contain *PRF* gene (codon-optimized), Kan^R^ |
| --- | --- |
| pCold I-PRF | pCold I, contain *PRF* gene (codon-optimized), Amp^R^ |
| △KshA | pET28a, contain *△KshA* gene, Kan^R^ |
| △KshB | pCold I, contain *△KshB* gene, Amp^R^ |
| △TDO | pET28a, contain *△TDO* gene, Kan^R^ |
| △PRF | pET28a, contain *△PRF* gene, Kan^R^ |
| pET28a-TeFdx | pET28a, contain *TeFdx* gene (codon-optimized), Kan^R^ |
| pET28a-DmFdx1 | pET28a, contain *DmFdx1* gene (codon-optimized), Kan^R^ |
| pET28a-DmFdx2 | pET28a, contain *DmFdx2* gene (codon-optimized), Kan^R^ |
| pET28a-PrFdx | pET28a, contain *PrFdx* gene (codon-optimized), Kan^R^ |
| pET28a-PsFdx1 | pET28a, contain *PsFdx1*gene (codon-optimized), Kan^R^ |
| pET28a-SyFdx | pET28a, contain *SyFdx* gene (codon-optimized), Kan^R^ |
| GST-DmFdx2 | pGEX-6p-3, contain *DmFdx2* gene (codon-optimized), Amp^R^ |
| GST | pGEX-6p-3, Amp^R^ |
| pETDuet-PRF-DmFdx2 | pETDuet, contain *PRF* and *DmFdx2* genes (codon-optimized), Amp^R^ |
| pETDuet-PRF-PsFdx1 | pETDuet, contain *PRF* and *PsFdx1* genes (codon-optimized), Amp^R^ |

**Table S3.** AD conversion rate of reductase and ferredoxin

| Reductase | Ferredoxin | | Conversion Rate (%) |
| --- | --- | --- | --- |
|  | Reiske-type  PsFdx1 | Plant-type  DmFdx2 |  |
| MTDO | **-** | **-** | 65.2 ± 1.16 |
| TDO | **-** | **-** | 54.8 ± 1.74* |
|  | **+** | **-** | 60.7 ± 0.35 |
|  | **-** | **+** | 64.3 ± 1.27 |
| MKshB | **-** | **-** | 43.2 ± 0.31 |
| KshB | **-** | **-** | 42.0 ± 1.29* |
|  | **+** | **-** | 49.6 ± 0.28 |
|  | **-** | **+** | 54.8 ± 1.02 |
| MPRF | **-** | **-** | 44.1 ±0.58 |
| PRF | **-** | **-** | 68.4 ± 0.39 |
|  | **+** | **-** | 80.6 ± 0.57 |
|  | **-** | **+** | 85.2 ± 1.63 |

Note: Data are shown as the mean ± SD from three independent experiments.

* data from *Zhu et al.*

**Table S4.** AD conversion rate of six Fdxs in KshA+PRF+Fdx catalytic reaction system

| Reductase | Ferredoxin | | Conversion Rate (%) |
| --- | --- | --- | --- |
|  | Reiske-type | Plant-type |  |
| PRF | PsFdx1 |  | 80.6 ± 0.57 |
|  | SyFdx |  | 70.3 ± 0.75 |
|  | PrFdx |  | 71.5 ± 0.67 |
|  |  | DmFdx2 | 85.2 ± 1.63 |
|  |  | DmFdx1 | 72.2 ± 0.29 |
|  |  | TeFdx | 73.2 ± 1.33 |

Note: Data are shown as the mean ± SD from three independent experiments.

**
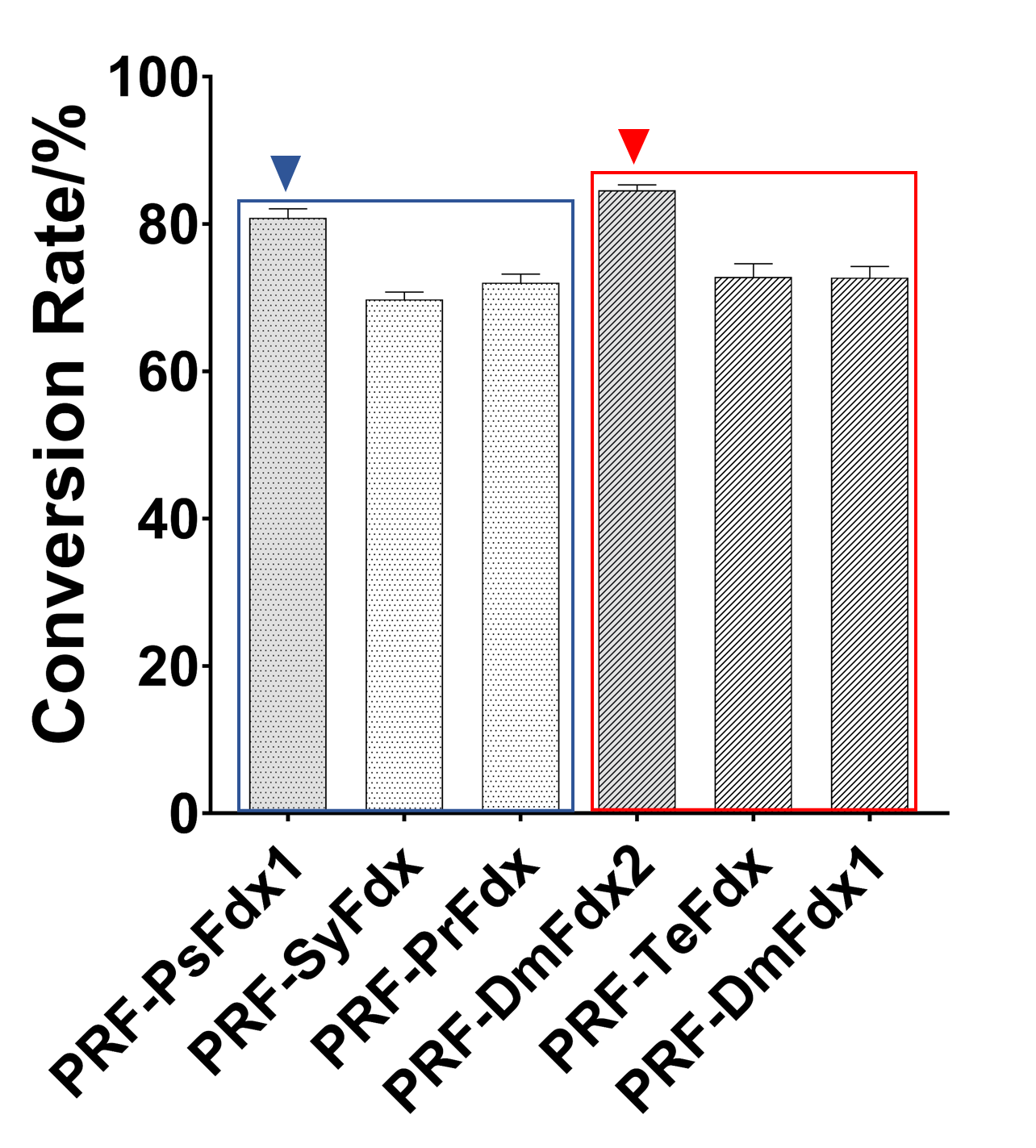
**

**Figure S1. Conversion rate of AD in enzyme-catalyzed reaction system after adding free Fdxs.** AD conversion rate of reductase PRF and six free ferredoxins of PsFdx1, SyFdx, PrFdx, DmFdx2, TeFdx, and DmFdx1 in KshA+PRF+Fdx catalytic reaction system. KshA+PRF+Rieske-type Fdxs and KshA+PRF+plant-type Fdxs are shown as slash (blue line frame) and dot (red line frame) filled, respectively.

**
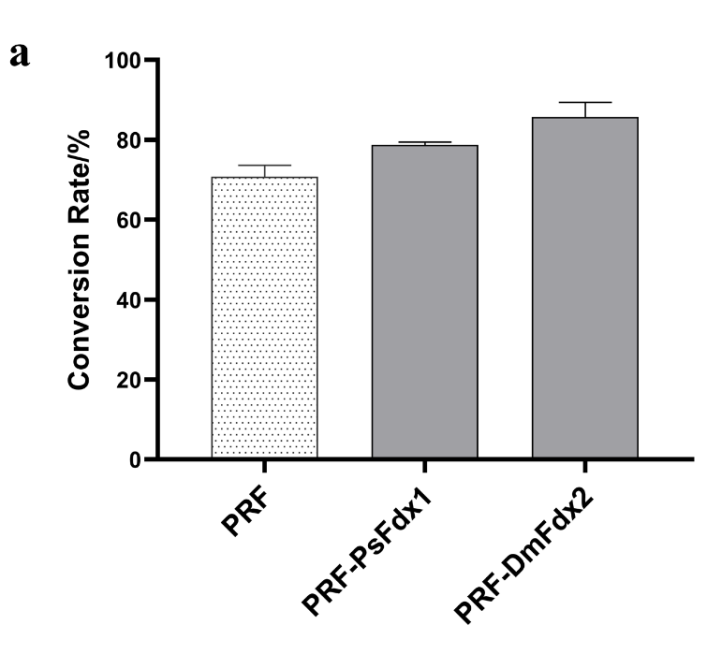

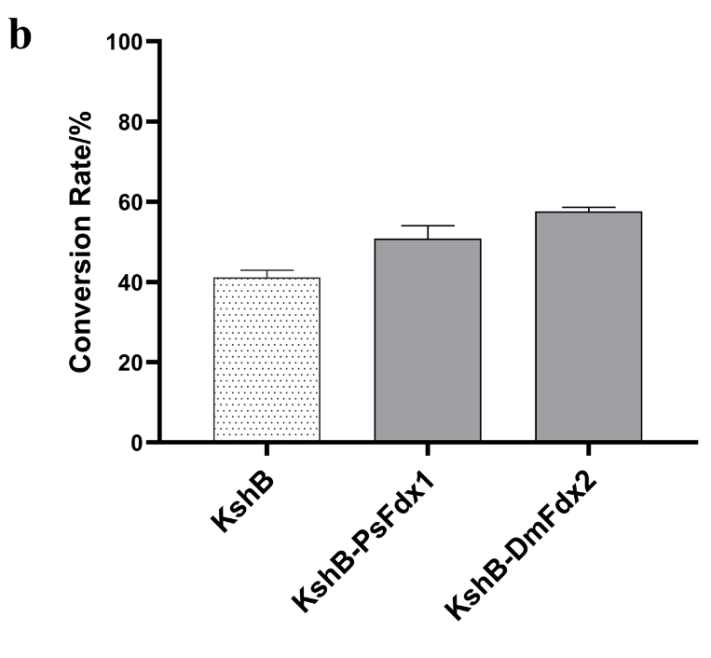
**

**
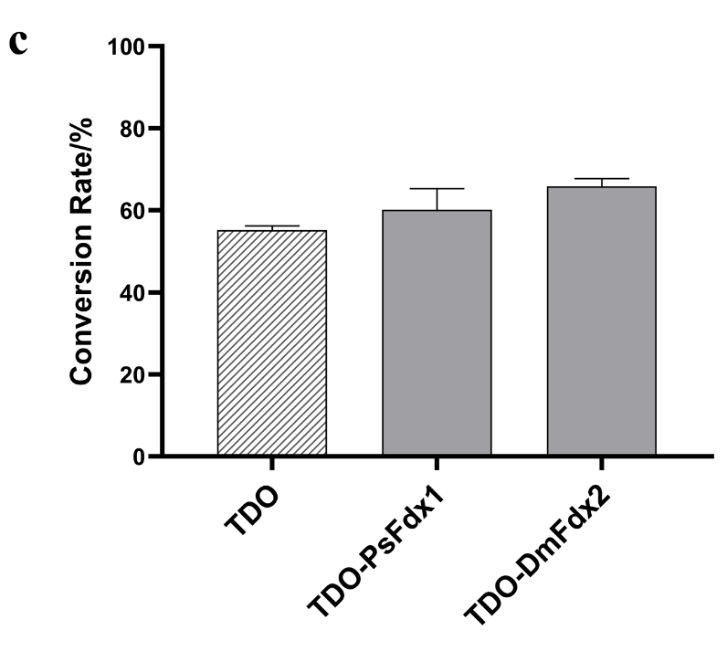
**

**Figure S2. Conversion rate of AD in multienzyme cascade catalysis adding free Fdxs.** AD conversion rate of reductase PRF(a), KshB(b), TDO(c) and free ferredoxin PsFdx1, DmFdx2 in KshA+reductase+Fdx catalytic reaction system.

**
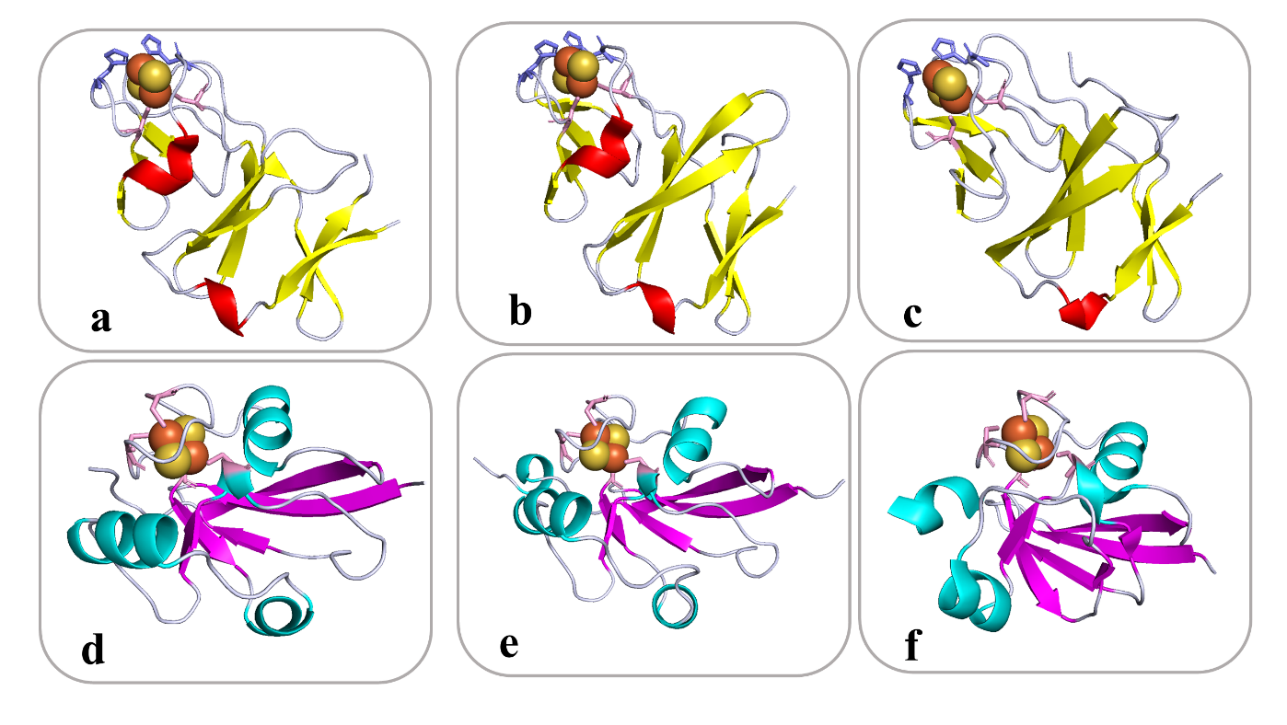
**

**Figure S3. Structural comparison of plant-type and Rieske-type Fdxs.** A cartoon representation of the overall structure of six Fdxs. The [2Fe-2S] clusters are represented by rods, and sulfur and iron are yellow and orange respectively. Cys and His coordinated with [2Fe-2S] clusters are represented as pink and purple sticks respectively. (a) SyFdx(PDB ID: 2I7F), (b) PrFdx(PDB ID: 1VCK) and (c) PsFdx1(PDB ID: 2E4P) are Rieske-type Fdxs. (d)DmFdx1, (e)DmFdx2 and (f)TeFdx(PDB ID: 5AUI) are plant-type Fdxs.

**
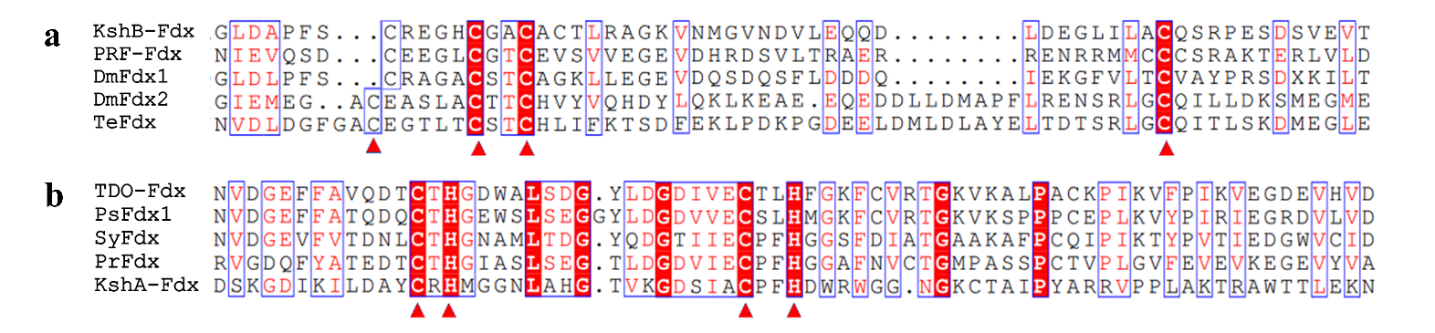
**

**
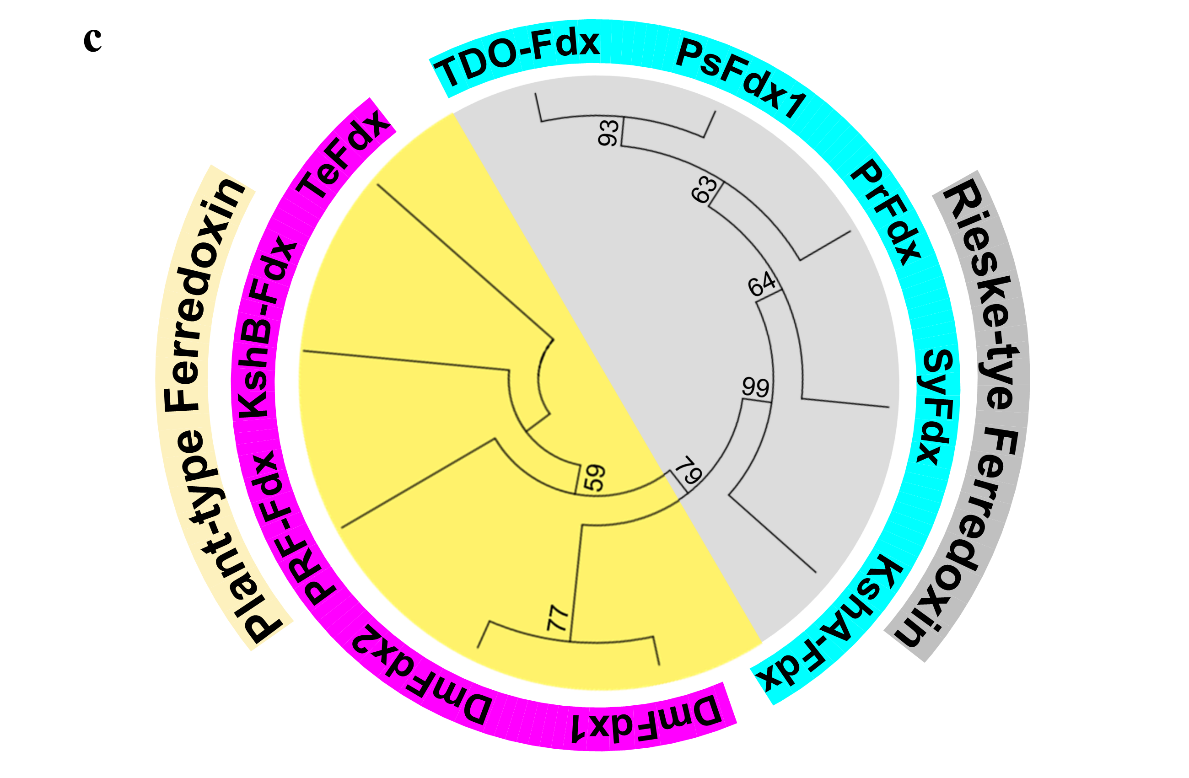
**

**Figure S4. Sequence alignment and** **phylogenetic tree analysis of [2Fe-2S] cluster domain of Fdxs and oxidoreductases.** (a) The sequence alignment of the 4Cys-coordinated [2Fe-2S] cluster domains of plant-type Fdxs. (b) The sequence alignment of the 2His- and 2Cys-coordinated [2Fe-2S] cluster domains of Rieske-type Fdxs. (c) The phylogenetic tree analysis of plant-type and Rieske-type sulfur cluster domains of Fdxs and oxidoreductases.


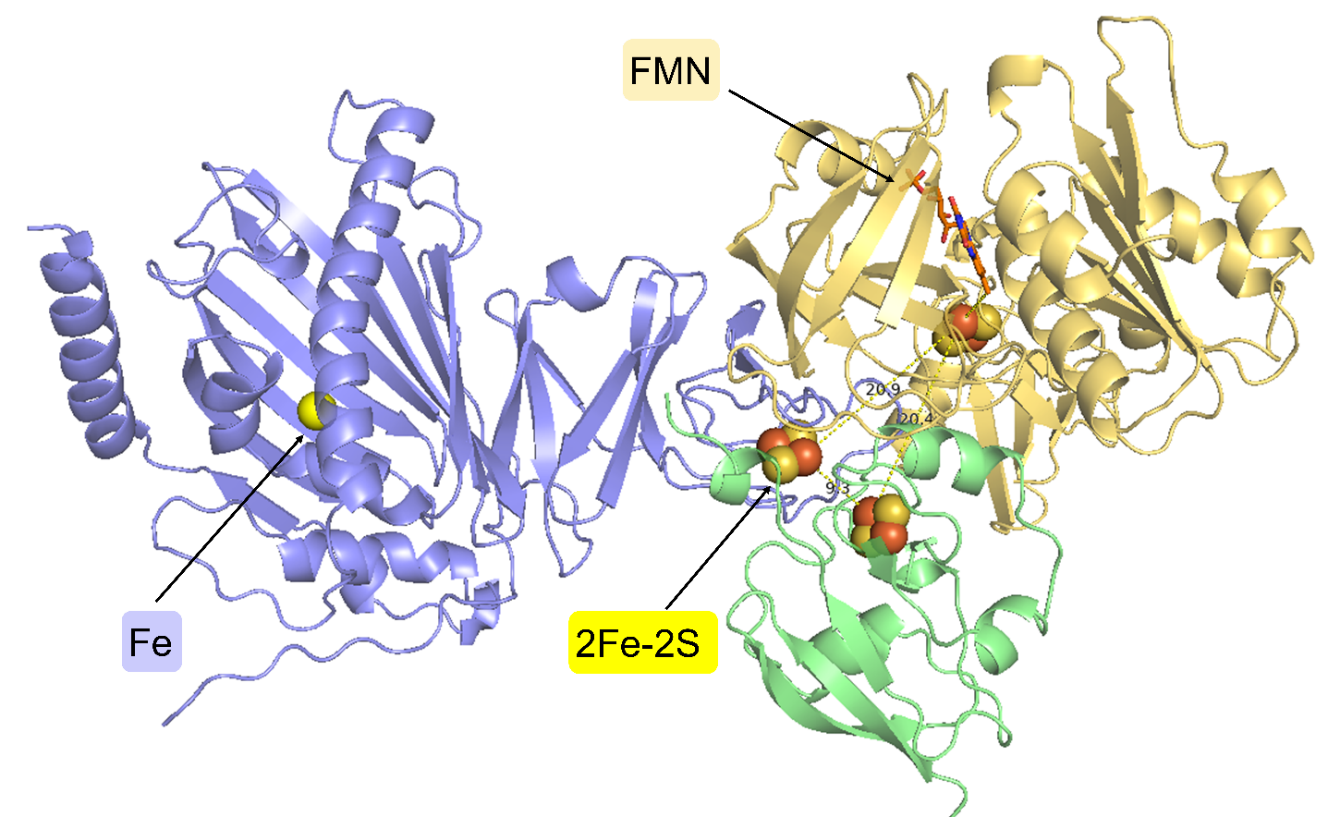


**Figure S5. Protein-protein docking of PRF, DmFdx2 and KshA complex**. The overall structure was shown in cartoon form and reductase PRF is shown in yellow, ferredoxin DmFdx2 is shown in green, and oxygenase KshA is shown in purple. The Fe atom in KshA appear as yellow spheres. The [2Fe-2S] clusters are displayed in a spherical shape, and sulfur and iron are displayed in yellow and orange respectively. The cofactor FMN in the PRF is displayed as sticks.
